# Efficacy of a Novel Intra-Articular Administration of Platelet-Rich Plasma One-Week Prior to Hyaluronic Acid versus Platelet-Rich Plasma Alone in Knee Osteoarthritis: A Prospective, Randomized, Double-Blind, Controlled Trial

**DOI:** 10.3390/jcm11113241

**Published:** 2022-06-06

**Authors:** Yung-Tsan Wu, Tsung-Ying Li, Kuei-Chen Lee, King Hei Stanley Lam, Chih-Ya Chang, Cheng-Kuang Chang, Liang-Cheng Chen

**Affiliations:** 1 Department of Physical Medicine and Rehabilitation, Tri-Service General Hospital, School of Medicine, National Defense Medical Center, Taipei 11490, Taiwan; crwu98@gmail.com (Y.-T.W.); doc31141@gmail.com (T.-Y.L.); juodo87@gmail.com (K.-C.L.); gradesboy@gmail.com (C.-Y.C.); 2 Integrated Pain Management Center, Tri-Service General Hospital, School of Medicine, National Defense Medical Center, Taipei 11490, Taiwan; 3 Department of Research and Development, School of Medicine, National Defense Medical Center, Taipei 11490, Taiwan; 4 Graduate Institute of Medical Sciences, National Defense Medical Center, Taipei 11490, Taiwan; 5 The Hong Kong Institute of Musculoskeletal Medicine, Hong Kong, China; drlamkh@gmail.com; 6 Department of Family Medicine, The Chinese University of Hong Kong, Hong Kong, China; 7 Department of Family Medicine, The University of Hong Kong, Hong Kong, China; 8 Center for Regional Anesthesia and Pain Medicine, Wan Fang Hospital, Taipei Medical University, Taipei 116, Taiwan; 9 Department of Physical Therapy and Assistive Technology, National Yang-Ming Chiao Tung University, Taipei 112, Taiwan; 10 Department of Radiology, Tri-Service General Hospital, School of Medicine, National Defense Medical Center, Taipei 11490, Taiwan; changlight3@gmail.com

**Keywords:** knee, osteoarthritis, platelet-rich plasma, hyaluronic acid, balance

## Abstract

Recent studies have suggested that the combined injection of platelet-rich plasma (PRP) and hyaluronic acid (HA) may have additive benefits for knee osteoarthritis over PRP alone, but there is insufficient evidence to support this combined injection. Moreover, the simultaneous injection of PRP and HA may offset the combined effect. Hence, the aim of this prospective, randomized, double-blind study was to assess their combined efficacy with a novel injection protocol. Forty-six study subjects with unilateral knee osteoarthritis were randomized to receive either a single-dose injection of HA (intervention group) or normal saline (control group) 1 week after a single-dose injection of leukocyte-poor PRP. The Western Ontario and McMaster Universities Osteoarthritis Index (WOMAC) and static balance and the risk of falls measured by Biodex Balance System were, respectively, the primary and secondary outcome measures. Evaluations were performed at baseline, 1 month, 3 months, 6 months, and 12 months post-injection. The intervention group exhibited significant declines in WOMAC pain, stiffness, and total scores, as well as static balance, compared to the control group (*p* < 0.05). These randomized double-blind control trials, with novel protocol of intra-articular injection of PRP 1-week prior to HA, provide greater symptom relief and improve static balance compared to PRP alone in patients with knee osteoarthritis.

## 1. Introduction

Osteoarthritis of the knee is a chronic degenerative disorder associated with the progressive breakdown of the articular cartilage in synovial joints, accompanied by the development of osteophytes, subchondral sclerosis, and cyst formation. It is the most common joint disease, with an annual incidence of 4.7% to 6.0%, and it is more common in female individuals than in male individuals (as high as 4:1) [1,2,3]. Furthermore, the impact of sex is more noticeable over the age of 50 years because the risk among female and male individuals is similar before the age of 50 years [4]. The clinical manifestations of knee osteoarthritis include pain, stiffness, effusion, deformity of the knee, and progressive limitation while performing daily activities. The prevalence of knee osteoarthritis is growing, and its consequences have a debilitating effect on the quality of life. In addition to the above symptoms, the mechanical receptors around the knee joint are more vulnerable due to osteoarthritis-induced anatomical changes. These are accompanied by declines in muscle strength, joint proprioception, and balance [5,6]. Therefore, the risk of falls is increased in patients with knee osteoarthritis, which is one of the most common causes of disability [7]. Knee osteoarthritis is primarily managed with either surgery or a non-surgical intervention. Non-surgical interventions include body weight reduction, physical therapy, administration of analgesics, and intraarticular injections with corticosteroids, hyaluronic acid (HA), or platelet-rich plasma (PRP). The main goal of non-surgical treatment is to modify the lifestyle, to relieve symptoms, to minimize disability, and to delay the progression of knee osteoarthritis. Surgery is generally reserved for patients who have very severe knee osteoarthritis or a poor response to non-surgical treatment [8].

Numerous studies have indicated that the injection of both HA and PRP improves cartilage restoration and decelerates the progress of knee osteoarthritis. HA, a cross-linked polysaccharide consisting of N-acetyl-D glucosamine and D-glucuronic acid, is abundant in the extracellular matrix of the connective tissues and synovial fluids. As there is a reduction in viscoelastic properties, resulting from decreased concentration and molecular weight of HA in patients with knee osteoarthritis, its injection is assumed to restore synovial fluid viscoelasticity, reduce synovial inflammation, enhance shock absorption in the knee joint, protect against cartilage damage, proteoglycan synthesis, and cartilage matrix alterations, as well as promote endogenous HA production [9,10]. Although the role of HA in the pathophysiology of osteoarthritis is complex, CD44-mediated effects of HA are shown to mainly contribute to the potential mechanism. Research shows that HA could bind to the CD44 receptor on chondrocytes and reduce interleukin-1β expression, leading to decreased activity of matrix metalloproteinase-1, 2, 3, 9, and 13 [11]. High molecular weight HA has better effects on knee osteoarthritis than low molecular weight HA because it has a higher viscosity, can delay the rate of metabolism, and has better anti-inflammatory effects for better protection and delaying degeneration of cartilage [12,13]. PRP consists of autologous concentrated platelets obtained from a patient’s own blood, which contains numerous growth factors. Given its biochemical and biological nature, PRP has been a focus of attention for the treatment of knee osteoarthritis. PRP can modulate the inflammatory response and regulate angiogenesis and cell differentiation to promote synovial cell proliferation and recovery of cartilage morphology [14]. Moreover, PRP can lubricate the joint by regulating endogenous HA synthesis [15]. Previous systematic reviews and meta-analyses have shown that, in comparison to HA, PRP produces greater reductions in pain and has more favorable effects on function restoration, for up to 12 months, in patients with knee osteoarthritis [16,17].

HA mainly restores the viscoelastic properties of synovial fluid, thereby reducing articular cartilage wear and pain, with limited biological effects on the regeneration of the impaired cartilage. In contrast, PRP can promote chondrocyte proliferation, cartilage synthesis, and increase the production of HA from native synoviocytes [14]. Furthermore, the addition of HA can induce the release of PRP growth factors [18]. Previous studies have reported that combination therapy with PRP and HA can further modify the inflammatory cytokines of the degenerative progression of chondrocytes, via certain mediators (CD44 and transforming growth factor β type II receptor), and correspondingly improve the regeneration of cartilage and decrease the inflammation in osteoarthritis [14,19]. Based on these findings, recent clinical studies have suggested that the combined injection of PRP and HA may have potential additive benefits for knee osteoarthritis than PRP alone because combined injection may take advantage of their dissimilar biological mechanisms (rheological properties of HA and regenerative potential of PRP) to enhance the activity of signal molecules, such as inflammatory molecules, catabolic enzymes, cytokines, and growth factors [20,21,22,23,24,25,26]. Nevertheless, there is a lack of compelling data to support this combined injection because, currently, published studies have methodological limitations and controversial results. In previous studies, simultaneous injection of PRP and HA may have offset the combined effect due to dilution of PRP growth factor and HA viscoelasticity. Moreover, these studies have mostly used subjective assessments and have not evaluated the effects of combined PRP and HA on balance and the risk of falls, which are important indicators of quality of life and the ability to perform daily activities. Thus, the aim of this prospective, randomized, double-blind study was to evaluate the effects of a PRP and HA combination therapy, with a novel injection protocol on pain, functional activity, balance, and the risk of falls in patients with knee osteoarthritis.

## 2. Materials and Methods

### 2.1. Study Design

**Figure 1:**
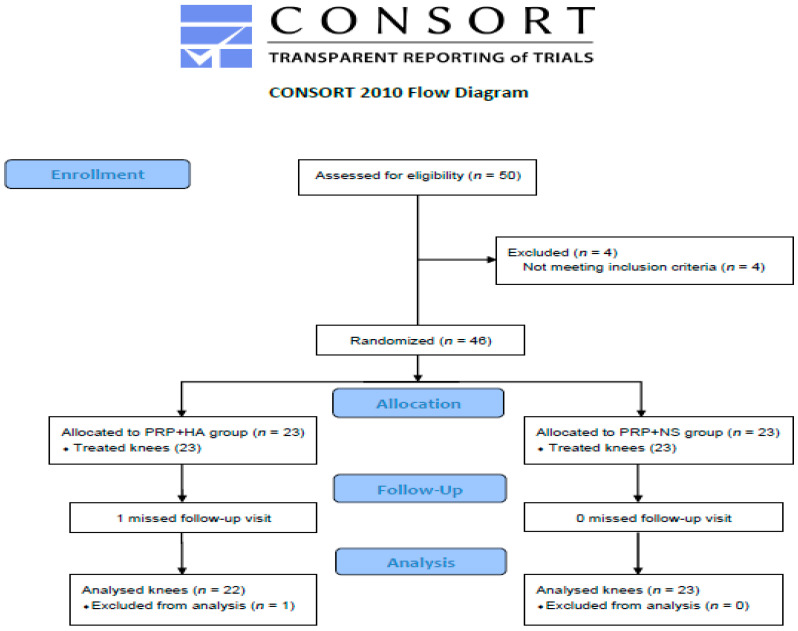
Study flow diagram.

This study was conducted between January 2018 and July 2021. The study protocol was reviewed and approved by the institutional review board of Tri-Service General Hospital, School of Medicine, National Defense Medical Center, Taipei, Taiwan (TSGHIRB No. 2-106-05-133, approved on 15 November 2017). The procedures followed were in accordance with the ethical standards of the committee on human experimentation (institutional and national) as well as with the Helsinki Declaration of 1975, as revised in 2000. All eligible study subjects provided written informed consent prior to enrollment. This clinical study was registered at ClinicalTrials.gov (NCT03290365, accessed on 19 September 2017). Fifty study subjects with unilateral knee osteoarthritis were eligible for study inclusion; among them, 46 were ultimately enrolled. The randomization sequence was generated with Microsoft Excel, and participants were randomized (1:1 ratio) into either the intervention or control group via block randomization (Figure 1).

### 2.2. Participants

Study subjects who were radiographically diagnosed with mild-to-moderate unilateral knee osteoarthritis (Ahlback stage I–III) were enrolled into the study. The stage of knee osteoarthritis was determined by a single radiologist. Study subjects were included if they were between 50 and 75 years of age, experienced pain in the affected knee for more than 6 months, and reported a pain intensity of at least 4 on the visual analogue scale during activity [27]. The exclusion criteria comprised the following: intra-articular knee injections 6 months prior to the study; anti-inflammatory medications 1 week prior to the study, intra-articular tumors; previous knee surgery, thrombocytopenia (platelet count < 150,000), coagulopathy, platelet dysfunction, known rheumatoid arthritis or autoimmune diseases, allergic to any contents of the HA or local anesthetics, significant effusion of the joint just before injection, and an inability to undergo balance testing.

### 2.3. PRP Preparation

Whole blood (7 mL) from each patient was obtained, by venipuncture, from the ante-cubital vein. Samples were collected in a PLTenus Plus Platelet Concentrate Separator (PLTenus, TCM Biotech International Corp., New Taipei City, Taiwan); anticoagulant citrate dextrose solution-A served as an anticoagulant without any activators. Blood samples were centrifuged at 500–1200 g for 8 min, and approximately 3.5–4 mL of leukocyte-poor PRP was subsequently isolated [28,29].

### 2.4. Intra-Articular Injection

A single physician, with 10 years of experience in intra-articular knee injections, performed all injections in this study. The injecting physician is not involved in assessing the efficacy of the injection to avoid bias. The study subjects were seated with their knee extended, and the needle was inserted into the joint space via a landmark-guided superlateral approach [30]. Study subjects in both groups received an initial single intra-articular injection of PRP. One week later, study subjects in the intervention and control groups were administered a single injection of HA (Hyafelic Uno, 2% cross-linked hyaluronan, 60 mg/3 mL Felixida Biotech Co., Ltd., Taipei, Taiwan), and normal saline (3 mL), respectively. Syringes containing HA and normal saline were identically marked; study subjects were also asked to turn their head away from the syringe during the injection so that they would be blind to group allocation. The injecting physician injected the HA and normal saline at the same average speed. Except for acetaminophen (500 mg, up to 4 g per day), other treatments (i.e., physical therapy, analgesics, other intraarticular injections) for knee osteoarthritis were not prescribed during the study period. The study subjects were instructed to record the frequency and dosage of acetaminophen taken.

### 2.5. Outcome Measurements

All outcomes were measured before injection and 1, 3, 6, and 12 months post-injection by the same investigator (not the injecting physician), who was blinded to group allocation.

Changes in primary and secondary outcomes, from pre-injection to 12 months post-injection, were assessed and compared between groups.

#### 2.5.1. Primary Outcome

The Western Ontario and McMaster Universities Osteoarthritis Index (WOMAC) is the most commonly utilized measurement for knee osteoarthritis. It consists of three subscales with a total of 24 items; these include 5 items for pain, 2 items for stiffness, and 17 items for function. We used the 10 cm Visual Analog Chinese version. The score for each item ranges from 0 (mildest) to 10 (most severe) points [31,32]. The WOMAC subscales were internally consistent, with Cronbach’s coefficient alpha of 0.91, 0.81, and 0.84, respectively, and the test-retest reliability of WOMAC was satisfactory, with intraclass correlation coefficients of 0.86, 0.68, and 0.89, respectively [33]. The minimal clinically important difference (MCID) was defined as a 12% to 18% reduction in WOMAC pain scores [34] and a 21.1% to 26% reduction in WOMAC function scores [35].

#### 2.5.2. Secondary Outcomes

The Biodex Balance System SD (Biodex Medical Systems, Shirley, NY, USA) is a commercially available balance device, which is used to assess static balance and the risk of falls [36,37]. In brief, study subjects stood barefoot with their arms resting on either side of their body; they were asked to maintain their equilibrium without handrail support and with their eyes open during the 20-s test. Each test was performed three times with a 10-s rest period, and the average value was used for statistical analyses. The platform settings were static in the balance assessment; the postural stability test mode contained the overall stability index (OSI), anterior-posterior stability index (APSI), and medial-lateral stability index (MLSI). Level 8, as well as levels 6 to 2, were used to evaluate the risk of falls, with lower scores indicating better balance control and lower fall risk.

### 2.6. Sample Size

We used a preliminary power analysis to calculate the sample size (G*power 3.1.9.2, UCLA, Los Angeles, CA, USA) [38], in order to reduce type II error. At least 42 participants were required to compare the intergroup difference in mean WOMAC values, at baseline and 12 months post-injection, according to the following parameters: power (1 − β) = 0.75; α = 0.05; effect size = 0.85.

### 2.7. Statistical Analysis

Statistical analyses were performed using IBM SPSS Statistics Version 22. Demo- graphic data were analyzed with the Mann–Whitney U test for continuous data and the Chi-square test or Fisher’s exact test for categorical data. Differences within groups were assessed with Friedman and Wilcoxon signed-rank post hoc tests, while differences between groups were evaluated with the Mann–Whitney U test. Statistical significance was set at *p* < 0.05.

## 3. Results

### 3.1. The Clinical and Demographic Characteristics of Study Subject

Forty-five study participants completed this study; one subject dropped out of the study due to personal reasons in the intervention group (Figure 1). There were 22 knees in the intervention group and 23 knees in the control group. Table 1 presents the clinical and demographic characteristics of the study subject, including age, sex, BMI, background chronic diseases, and osteoarthritis severity in the Ahlback stages. The mean age was 62.2 ± 1.5 and 61.3 ± 1.4 years, and the mean symptom duration was 34.6 ± 6.3 and 31.3 ± 7.3 months in the intervention and control groups, respectively. Before the intervention, neither group differed significantly. No side effects resulting from the injections were observed, and no additional conservative treatments were required during the follow-up period.

### 3.2. The Effect of WOMAC

**Figure 2:**
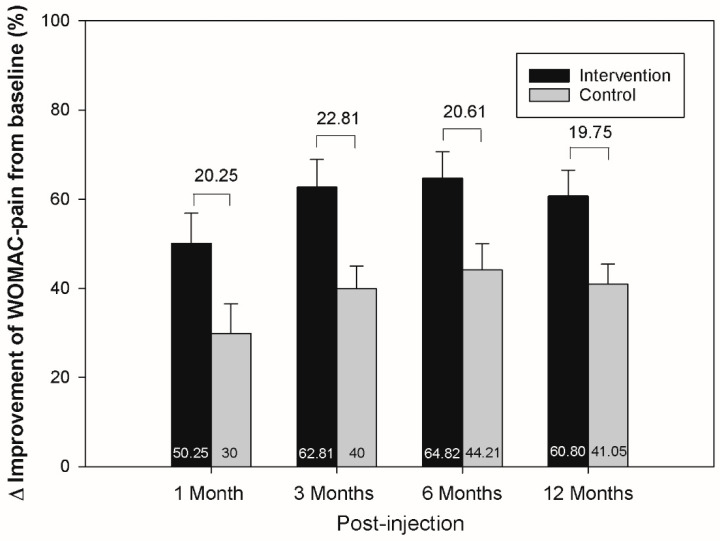
Differences (%) in Western Ontario and McMaster Universities Osteoarthritis Index (WOMAC) pain scores, between post-injection and baseline, in both groups at each time point. There were greater improvements in the intervention group. The intergroup differences ranged from 19.75% to 22.81%. The upper whiskers represent standard errors.

Both groups exhibited significant improvements in WOMAC scores at all follow-up assessments compared to baseline (*p* < 0.05) (Table 2). The intervention group exhibited significantly greater improvements than the control group in WOMAC pain, stiffness, and total scores at the majority of follow-up assessments (all *p* < 0.05); exceptions included the assessments at 1 (WOMAC pain, stiffness, and total scores), 3 (WOMAC stiffness scores), and 6 months (WOMAC total scores). While a greater reduction in WOMAC function scores was observed in the intervention group compared to the control group, this did not reach statistical significance. Differences in WOMAC pain scores between groups ranged from 19.75% to 22.81%; this exceeded the MCID (Figure 2). Table 3 shows the proportion of study subjects who met the MCID of the WOMAC pain and function scores, and the difference between groups was not significant throughout the follow-ups.

### 3.3. The Effect of Balance and the Risk of Falls

The results of the static balance and risk of fall analyses are shown in Table 4. Both groups exhibited significant improvements, compared to the baseline, at most follow-up time points; exceptions included assessments at 3 (balance-OSI), 3–12 (balance-APSI), and 1–12 (balance-MLSI) months. Significantly greater reductions in balance-OSI (3–12 months) and balance-MLSI (3–6 months) were observed in the intervention group compared to the control group. The intervention group also exhibited a tendency toward greater reductions in balance-APSI scores than the control group over longer follow-up assessments. While greater reductions in the risk of falls were observed in the intervention group, there were no significant differences compared with the control group.

## 4. Discussion

This is the first prospective, randomized, double-blind study to simultaneously evaluate the efficacy of a PRP and HA combination therapy with a novel injection protocol on symptoms, balance, and the risk of falls in patients with knee osteoarthritis. The results indicated that this combination therapy significantly improved pain, stiffness, and static balance compared to PRP alone. While the combination therapy also resulted in more improvements in function and reductions in the risk of falls, compared to the administration of PRP alone, these did not reach statistical significance.

The additive effect of PRP and HA may be associated with an improved ability to augment various healing processes with regenerative and anti-inflammatory effects. Indeed, recent experimental studies support their additive mechanisms. The anti-inflammatory effects of both PRP and HA may be partially attributed to the reduction in tumor necrosis factor-α. However, Sundman et al. [39] have also reported distinct anti-inflammatory mechanisms; interleukin-6 is only reduced by HA, while PRP decreases metalloproteinases. Laboratory studies have also shown a 335% increase in the migration of fibroblasts, with the combined use of PRP and HA, compared to HA alone [40]. These observations support the use of the combination therapy to promote regenerative effects, as cell migration has an essential role in tissue regeneration. Iio et al. [18] reported that the addition of HA can induce the release of PRP growth factor, and PRP can also enhance the secretion of HA [41]. Chen et al. [19] demonstrated that the combination of HA and PRP could additively promote cartilage regeneration and inhibit osteoarthritis inflammation by reducing the expression of inflammatory genes, as well as related chemokines and cytokines genes, compared with HA alone. Other studies have also demonstrated that the combination of PRP and HA can significantly increase the chondrocyte proliferation rate and glycosaminoglycan content, while also decreasing apoptosis and cartilage damage [42,43,44]. Thus, based on their different biological mechanisms and additive effects, the combined use of PRP and HA deserves consideration as a novel and effective treatment for knee osteoarthritis, especially in patients with an advanced severe stage because the current evidence-based effect of PRP or HA alone injection, for severe knee osteoarthritis, is limited [16,17]. However, our results, as with all other published studies, cannot be generalized to severe osteoarthritis populations. Thus, further research involving study subjects with severe disease is encouraged.

The combined injection of PRP and HA was first reported in 2015; however, its adjunctive effects, compared with PRP injection, alone, remain unclear [20,21,22,23,24,25,26]. The pioneering study utilized a retrospective design with a 6-month follow-up. Abate et al. [20] reported that the combination therapy had an equivalent efficacy compared to PRP alone; a series of three injections were administered, spaced by 1-week intervals. Lana et al. [21] conducted a double-blind trial with a 1-year follow-up. They found that the combination therapy (three injections, spaced by 2-week intervals) yielded better outcomes at 3 months. Palco et al. [25] conducted a retrospective study with a 1-year follow-up and reported greater improvements in knee mobility and function with the combination therapy (three injections, spaced by 2-week intervals). Recently, Sun et al. [26] performed a prospective, randomized-controlled, observer-blinded study with 6 months of follow-up. Their results indicated that combined injection, with single PRP and HA, resulted in a significant reduction in the VAS score at 6 months, whereas PRP, alone, was more effective than the combined injection in reducing VAS and WOMAC-pain and WOMAC-stiffness scores at 1-month post-injection. Furthermore, a recent meta-analysis concluded that the combination therapy improved symptoms and reduced disability at 6 months in comparison to PRP alone [45].

In contrast to the aforementioned studies, other studies have revealed that the combined injection has no additive benefits. In their retrospective study with 12 months of follow-up, Guo et al. [22] described that the combined injection (three injections, 1 week apart) is not superior to PRP alone. Jacob et al. [23] conducted a comparative study, with 6 months of follow-up, and found that the combination therapy of single PRP with HA (no details provided on HA composition or how it was combined with PRP) is not superior to PRP alone. In a 1-year double-blind randomized clinical study, Nasser et al. [24] also claimed that blending HA with PRP (five injections, 1 week apart) had no additional advantage compared with PRP alone. A recent systematic review further concluded that combination injection is not superior to PRP alone [46]. The variable effectiveness in published studies may be due to different methodology, including inclusion/exclusion criteria, injection protocols, study designs, variable concentrations of PRP and different HA products, etc. Actually, there were only two double-blind randomized clinical studies [21,24], and most previous studies did not use equal injection volume between the groups, which means different capsular distension can affect the outcomes [20,23,25,26]. Nevertheless, the present study is a well-designed double-blind randomized study, and its results support the adjunctive effect of HA, when combined with PRP, for the treatment of patients with knee osteoarthritis.

The issue worth exploring is the order in which PRP and HA are injected. All previous studies, which have evaluated this combination therapy, have injected both agents simultaneously. However, a limited number of studies have investigated the potential disadvantages of such a technique [42,47,48]. Dallari et al. [47] reported that PRP, alone, was more effective than the combination therapy for pain relief, within 6 months post-injection, in patients with hip osteoarthritis. They commented that the simultaneous injection of PRP and HA (total of 9 mL) likely resulted in the dilution of PRP growth factors; this would have led to extreme capsular distension and increased pain. Patel et al. [48] echoed these findings and advised that PRP should be administered 1 month prior to HA. They reasoned that this injection sequence would avoid the possibility of antagonistic effects, while providing an optimal additive benefit. Russo et al. [42] found that a HA concentration below 1% was associated with a notable decrease in viscoelastic properties after the addition of PRP; this was attributed to a change in the rheological profile of HA via a dilution effect. Studies have shown that almost 95% of the platelet growth factors are secreted from the PRP preparations within the first hour post injection, although small amounts of growth factors may still be released during the residual life span of platelets (8–10 days) [49,50]. Therefore, in our study, we administered the PRP injection 1 week prior to the HA injection; thus, this may be the first study to apply this novel injection protocol for the treatment of knee osteoarthritis, and the results were similar, or even superior, to those of previous studies. Although the therapeutic effect of spacing between injections from different studies’ designs cannot be directly compared, we would like to project the necessity of further study to explore this worthwhile issue.

Chronic knee pain can result in substantial declines in balance and strength in patients with knee osteoarthritis [5], and an increased pain severity is also associated with a higher risk of falls [7]. There is currently a lack of evidence pertaining to the effects of PRP on balance and the risk of falls in patients with knee osteoarthritis. Sun et al. [51] reported clinical improvements in pain and balance after HA injection. Likewise, Khalaj et al. [52] observed significant improvements in postural stability and the risk of falls after HA injection. These results suggest that static balance and the risk of falls can improve with pain relief. Although both groups in our study have noticed significant improvement in WOMAC function scores and a reduction in the risk of falls compared to their baseline, the between-group differences were not statistically significant. Our findings contrast with the results of previous studies [21,53,54], and these discrepancies may be due to multiple factors. First, balance control is a complex function, which requires the integration of proprioception, visual and vestibular systems, and muscle coordination [55]. While accidental falls and impaired balance most often occur during dynamic activities, they rarely occur during static activities [56]. Therefore, future studies should consider the use of dynamic balance tests, in addition to static balance tests, to examine the risk of falls. Second, pain relief does not necessarily lead to improvements in balance, proprioception, or strength in patients with knee osteoarthritis. Indeed, Hassan et al. [57] reported a lack of improvement in static postural stability 1 h post-injection; however, a significant reduction in pain was observed. Similarly, another study found no significant improvements in proprioception, although viscosupplementation was able to improve knee strength and WOMAC pain, stiffness, and function scores [58]. Wu et al. [27] did not observe significant improvements in muscle strength with the injection of PRP; however, they noted significant pain relief. Third, there may be potential compensation via a patient’s asymptomatic knee. As we only included patients with a unilateral symptomatic knee, the risk of falls related to reduced proprioception, balance, and strength may have been negated by the asymptomatic knee. This may also have explained the lack of significant differences in WOMAC function scores between groups. Fourth, pain alleviation may not improve certain dysfunctions caused by osteoarthritis progression; these may require additional interventions, such as strength and balance training, to improve function and reduce the risk of falls [59]. Nevertheless, this study is the first to demonstrate that the combination therapy can significantly improve static balance compared with PRP alone. It may be worthwhile to pursue further research in the same field.

Some limitations are acknowledged in the present study. First, we did not investigate the mechanism of combined PRP and HA therapy. Second, the sample size was relatively small, and most of the enrolled study subjects were female individuals because we included study subjects who were more than 50 years old. Hence, this may not be generalized to the entire population, although the sex ratio in this study is close to that reported in a previous study [3]. Future studies with larger sample sizes and equal numbers of male and female study subjects are required to validate our results. Third, PRP composition, including concentrations of platelets, white blood cells, and levels of cytokine/growth factors was not measured in our study. Although PLTenus Plus Platelet Concentrate Separator has been widely used in published studies [28,29,60], this limitation should be considered with caution. Fourth, even though all syringes were identical, the operator administering the injections was able to identify the injectate; this was attributed to differences in tactile sensation when depressing the plunger for the injection of HA versus normal saline. Nevertheless, the outcome assessor was blind to group allocation, and none of the participants were able to differentiate between the injectates due to the use of the same volume, injection speed, and schedule in both groups. Thus, we believe that the double-blinding in our study was adequate and did not affect the study outcomes. Fifth, WOMAC’s subjective questions without objective measurement, such as biochemical markers, fail to comprehensively investigate the symptom severity. Moreover, the simultaneous assessment of both dynamic balance and strength would have provided a more comprehensive evaluation of the efficacy of our intervention. Finally, we suggest that future studies compare the effects of different injection sequences and determine the optimal dosages of PRP and HA required to achieve the best therapeutic effect.

## 5. Conclusions

The present study provides a combined injection of PRP and HA, with the novel protocol of intra-articular injection of PRP 1-week prior to HA, providing greater symptom relief and improving static balance more effectively than PRP, alone, in patients with knee osteoarthritis. However, there is no difference between treatment groups in the percentage of patients that experienced a clinically significant outcome improvement.

## Figures and Tables

**Table 1:** Baseline demographic and clinical characteristics of study subjects.

	Intervention (PRP + HA) Group (*n* = 22)	Control (PRP + NS) Group (*n* = 23)	^a^*p* Value
Gender, *n* (%)			0.766
Female	18 (81.8)	18 (78.3)	
Male	4 (18.2)	5 (21.7)	
Age (year) ± SE (range)	62.2 ± 1.5 (50–74)	61.3 ± 1.4 (50–75)	0.569
BMI (kg/m^2^)	26.3 ± 0.9	25.5 ± 0.7	0.683
DM (%)	1 (4.5)	3 (13.0)	0.608
Hypertension (%)	11 (50.0)	5 (21.7)	0.065
Lesion site, *n* (%)			0.449
Left	9 (40.9)	12 (52.2)	
Right	13 (59.1)	11 (47.8)	
Duration (month) ± SE (range)	34.6 ± 6.3 (6–120)	31.3 ± 7.3 (6–120)	0.506
Ahlback Stage			0.896
I	12 (54.5)	13 (56.5)	
II	5 (22.7)	6 (26.1)	
III	5 (22.7)	4 (17.4)	
VAS (SE)	5.5 ± 0.2	5.7 ± 0.2	0.404
Lequesne index (SE)	11.9 ± 0.7	10.9 ± 0.5	0.308

PRP = Platelet-rich Plasma; HA = Hyaluronic acid; NS = Normal saline; BMI = Body mass index; DM = Diabetes mellitus; VAS = Visual analog scale; SE = standard error. ^a^ Mann–Whitney *U* Test, Chi-square test or Fishers exact test.

**Table 2:** Comparison of all WOMAC scores between both groups.

	Intervention (PRP + HA) Group (*n* = 22) Mean ± SE Mean Difference ± SE		*^a^ p* value	Control (PRP + NS) Group (*n* = 23) Mean ± SE Mean Difference ± SE		^a^*p* Value	^b^*p* Value
WOMAC (pain)	19.9 ± 1.4			19.0 ± 1.1			0.873
Month 1	10.0 ± 1.4	−10.0 ± 1.5	<0.001	13.3 ± 1.1	−5.7 ± 1.3	0.001	0.062
Month 3	7.4 ± 1.2	−12.5 ± 1.5	<0.001	11.4 ± 1.0	−7.6 ± 1.1	<0.001	0.017
Month 6	7.1 ± 1.2	−12.9 ± 1.4	<0.001	10.6 ± 1.1	−8.4 ± 1.2	<0.001	0.020
Month 12	7.8 ± 1.3	−12.1 ± 1.4	<0.001	10.8 ± 1.1	−7.8 ± 0.9	<0.001	0.049
WOMAC (stiffness)	8.5 ± 0.5			7.4 ± 0.4			0.107
Month 1	5.1 ± 0.7	−3.4 ± 0.7	0.001	4.9 ± 0.5	−2.4 ± 0.6	0.002	0.551
Month 3	3.4 ± 0.6	−5.1 ± 0.7	<0.001	4.1 ± 0.6	−3.2 ± 0.7	0.001	0.083
Month 6	2.9 ± 0.5	−5.6 ± 0.7	<0.001	4.4 ± 0.5	−3.0 ± 0.5	<0.001	0.006
Month 12	2.5 ± 0.5	−6.0 ± 0.6	<0.001	3.7 ± 0.5	−3.7 ± 0.5	<0.001	0.006
WOMAC (function)	67.1 ± 5.1			60.5 ± 4.2			0.207
Month 1	40.9 ± 5.5	−26.3 ± 5.4	0.001	39.8 ± 3.4	−20.7 ± 2.8	<0.001	0.910
Month 3	29.6 ± 4.6	−37.6 ± 5.2	<0.001	36.8 ± 3.5	−23.7 ± 3.3	<0.001	0.112
Month 6	25.4 ± 3.9	−41.7 ± 4.2	<0.001	28.4 ± 3.0	−32.1 ± 3.7	<0.001	0.102
Month 12	25.4 ± 3.8	−41.8 ± 4.5	<0.001	31.1 ± 3.4	−29.4 ± 4.2	<0.001	0.080
WOMAC (total)	95.5 ± 6.5			86.9 ± 5.1			0.237
Month 1	55.9 ± 7.3	−39.6 ± 7.3	<0.001	58.1 ± 4.6	−28.8 ± 3.8	<0.001	0.433
Month 3	40.3 ± 6.1	−55.2 ± 6.9	<0.001	52.4 ± 4.6	−34.5 ± 4.2	<0.001	0.040
Month 6	35.3 ± 5.3	−60.2 ± 5.7	<0.001	43.3 ± 4.1	−43.6 ± 4.7	<0.001	0.051
Month 12	35.6 ± 5.4	−59.9 ± 5.9	<0.001	45.6 ± 4.3	−41.3 ± 4.5	<0.001	0.021

PRP = Platelet-rich Plasma; HA = Hyaluronic acid; NS = Normal saline; WOMAC = Western Ontario and McMaster Universities Arthritis Index; SE = Standard error. ^a^ Friedman test with Wilcoxon Signed-Rank post hoc analysis (each time-points versus baseline). ^b^ Mann–Whitney *U* Test (mean difference, intergroup).

**Table 3:** Proportion of study subjects meeting the MCID of WOMC between groups.

	Intervention (PRP + HA) Group (*n* = 22)	Control (PRP + NS) Group (*n* = 23)	^a^*p* Value
	*n* (%)	*n* (%)	
WOMAC (pain)			
Month 1	17 (77.3)	17 (73.9)	0.793
Month 3	20 (90.9)	17 (73.9)	0.243
Month 6	21 (95.5)	20 (87.0)	0.608
Month 12	22 (100)	21 (91.3)	0.489
WOMAC (function)			
Month 1	14 (63.6)	16 (69.6)	0.673
Month 3	19 (86.4)	19 (82.6)	0.728
Month 6	20 (90.9)	22 (95.6)	0.608
Month 12	21 (95.5)	20 (87.0)	0.608

PRP = Platelet-rich Plasma; HA = Hyaluronic acid; NS = Normal saline; WOMAC = Western Ontario and McMaster Universities; MCID = minimal clinically important difference (>18% reduction in WOMAC-pain scores; >26% reduction in WOMAC-function scores). ^a^ chi-square test or Fisher’s Exact Test.

**Table 4:** Comparison of all balance and risk of fall analyses between both groups.

	Intervention (PRP + HA) Group (*n* = 22) Mean ± SE Mean Difference ± SE		^a^*p* Value	Control (PRP + NS) Group (*n* = 23) Mean ± SE Mean Difference ± SE		^a^*p* Value	^b^*p* Value
Balance-OSI	0.69 ± 0.04			0.65 ± 0.08			0.255
Month 1	0.54 ± 0.04	−0.15 ± 0.03	<0.001	0.58 ± 0.07	−0.07 ± 0.06	0.046	0.240
Month 3	0.44 ± 0.03	−0.25 ± 0.03	<0.001	0.51 ± 0.05	−0.15 ± 0.07	0.056	0.020
Month 6	0.43 ± 0.04	−0.26 ± 0.03	<0.001	0.52 ± 0.05	−0.14 ± 0.06	0.027	0.030
Month 12	0.40 ± 0.03	−0.29 ± 0.03	<0.001	0.47 ± 0.05	−0.18 ± 0.08	0.025	0.019
Balance-APSI	0.52 ± 0.03			0.52 ± 0.07			0.413
Month 1	0.38 ± 0.03	−0.14 ± 0.02	<0.001	0.41 ± 0.05	−0.11 ± 0.05	0.037	0.108
Month 3	0.35 ± 0.03	−0.17 ± 0.03	<0.001	0.39 ± 0.04	−0.13 ± 0.07	0.135	0.114
Month 6	0.33 ± 0.03	−0.19 ± 0.03	<0.001	0.39 ± 0.04	−0.12 ± 0.06	0.087	0.073
Month 12	0.32 ± 0.02	−0.20 ± 0.03	<0.001	0.39 ± 0.05	−0.13 ± 0.06	0.074	0.097
Balance-MLSI	0.36 ± 0.03			0.32 ± 0.05			0.127
Month 1	0.29 ± 0.04	−0.07 ± 0.03	0.025	0.30 ± 0.06	−0.03 ± 0.07	0.064	0.600
Month 3	0.22 ± 0.02	−0.15 ± 0.03	<0.001	0.28 ± 0.05	−0.04 ± 0.06	0.384	0.023
Month 6	0.25 ± 0.03	−0.11 ± 0.03	0.001	0.30 ± 0.03	−0.03 ± 0.05	0.765	0.042
Month 12	0.22 ± 0.03	−0.14 ± 0.04	0.001	0.25 ± 0.03	−0.07 ± 0.06	0.262	0.143
Risk fall-6 level	2.3 ± 0.2			2.1 ± 0.2			0.532
Month 1	1.9 ± 0.2	−0.4 ± 0.1	0.001	1.9 ± 0.2	−0.2 ± 0.1	0.030	0.180
Month 3	1.6 ± 0.1	−0.7 ± 0.1	<0.001	1.7 ± 0.2	−0.4 ± 0.1	0.004	0.149
Month 6	1.5 ± 0.1	−0.8 ± 0.1	<0.001	1.6 ± 0.2	−0.5 ± 0.2	0.005	0.334
Month 12	1.4 ± 0.1	−0.9 ± 0.2	<0.001	1.4 ± 0.2	−0.8 ± 0.1	<0.001	0.829
Risk fall-8 level	1.3 ± 0.1			1.1 ± 0.1			0.407
Month 1	1.1 ± 0.1	−0.1 ± 0.04	0.004	1.0 ± 0.1	−0.1 ± 0.04	0.020	1.000
Month 3	1.0 ± 0.1	−0.3 ± 0.07	0.002	1.0 ± 0.1	−0.1 ± 0.05	0.005	0.184
Month 6	1.0 ± 0.1	−0.2 ± 0.07	0.003	1.0 ± 0.1	−0.1 ± 0.05	0.005	0.439
Month 12	0.9 ± 0.1	−0.4 ± 0.10	0.001	0.9 ± 0.1	−0.3 ± 0.05	<0.001	0.323

PRP = Platelet-rich Plasma; HA = Hyaluronic acid; NS = Normal saline; SE = Standard error; OSI = overall stability index; APSI = anterior-posterior stability index; MLSI = medial-lateral stability index. ^a^ Friedman test with Wilcoxon Signed-Rank post hoc analysis (each time-points versus baseline). ^b^ Mann–Whitney *U* Test (mean difference, intergroup).

## Data Availability

The data presented in this study are available on request from the corresponding author.
